# A Deep Learning-Based Sensing System for Identifying Salmon and Rainbow Trout Meat and Grading Freshness for Consumer Protection

**DOI:** 10.3390/s25206299

**Published:** 2025-10-11

**Authors:** Hong-Dar Lin, Jun-Liang Chen, Chou-Hsien Lin

**Affiliations:** 1 Department of Industrial Engineering and Management, Chaoyang University of Technology, Taichung 413310, Taiwan; s10815615@cyut.edu.tw; 2 Department of Civil, Architectural, and Environmental Engineering, The University of Texas at Austin, Austin, TX 78712-0273, USA; chslin@utexas.edu

**Keywords:** food fraud, fish meat classification, freshness grading, deep learning, DenseNet121, transfer learning

## Abstract

Seafood fraud, such as mislabeling low-cost rainbow trout as premium salmon, poses serious food safety risks and damages consumer rights. To address this growing concern, this study develops a deep learning-based, smartphone-compatible sensing system for fish meat identification and salmon freshness grading. By providing consumers with real-time, image-based verification tools, the system supports informed purchasing decisions and enhances food safety. The system adopts a two-stage design: first classifying fish meat types, then grading salmon freshness into three levels based on visual cues. An improved DenseNet121 architecture, enhanced with global average pooling, dropout layers, and a customized output layer, improves accuracy and reduces overfitting, while transfer learning with partial layer freezing enhances efficiency by reducing training time without significant accuracy loss. Experimental results show that the two-stage method outperforms the one-stage approach and several baseline models, achieving robust accuracy in both classification and grading tasks. Sensitivity analysis demonstrates resilience to blur and camera tilt, though real-world adaptability under diverse lighting and packaging conditions remains a challenge. Overall, the proposed system represents a practical, consumer-oriented tool for seafood authentication and freshness evaluation, with potential to enhance food safety and consumer protection.

## 1. Introduction

Seafood fraud, especially the mislabeling of lower-value species as premium products, remains a widespread problem, compromising food safety, consumer trust, and regulatory oversight [1,2]. A common example is the substitution of rainbow trout for salmon in supermarkets and sushi restaurants, where visual similarities can easily mislead consumers. This deception poses health risks, as rainbow trout is more prone to parasitic contamination and is unsafe for raw consumption [3]. Additionally, undisclosed use of genetically modified or farmed fish raises further public health concerns and violates consumers’ right to informed choices.

Salmon is widely consumed in dishes like sashimi, sushi, and steaks, with rising consumer demand. As a migratory fish, it lives in seawater and spawns in freshwater, unlike rainbow trout, a freshwater species with a higher parasite risk, making it unsuitable for raw consumption. However, some vendors fraudulently substitute lower-cost rainbow trout for premium salmon, posing food safety and consumer rights concerns. To address this, the study proposes a smartphone-based system that distinguishes salmon from rainbow trout and evaluates salmon freshness. Consumers can upload fish images to a cloud server and receive real-time classification results, supporting safer and more informed purchasing decisions.

Salmon is commonly presented to consumers in several forms, such as steaks, fillets, and sashimi. These cut types differ in appearance and texture, offering important visual cues that help distinguish between salmon and similar species like rainbow trout. Because these consumer-ready presentations are the basis for both authenticity verification and freshness assessment, this study focuses on analyzing salmon in these forms while developing a practical, smartphone-compatible classification and grading system.

### 1.1. Visual Similarities in Salmon and Rainbow Trout Meat

Salmon and rainbow trout are both popular fish with similar appearances, but their meat differs in several key aspects, including biology, habitat, nutritional content, taste, texture, and suitability for certain preparations. Visual identification of salmon and rainbow trout meat can help consumers distinguish between the two, especially to avoid mislabeling or fraud. As summarized in Table 1, salmon typically shows vibrant pink to deep orange-red flesh with distinct white fat lines, a glossy texture, and thicker, more robust cuts that stay bright when fresh. Rainbow trout is usually paler (light pink to light orange), with faint marbling, a softer, less glossy texture, and thinner, more delicate fillets; when less fresh, its color often dulls toward grayish or whitish tones. Because some vendors may dye trout to resemble salmon, consumers should rely on multiple visual cues, especially marbling and texture, rather than color alone.

### 1.2. Visualization of Salmon Freshness

Freshness in salmon is closely tied to visual attributes such as flesh color, brightness, and marbling. These visible features serve as primary cues for both consumers and inspectors when judging quality. Unlike chemical or microscopic measures, which require laboratory equipment, visual cues can be quickly assessed through images, making them particularly relevant for consumer-facing systems. This subsection highlights the significance of visual indicators as the foundation for image-based freshness grading in this study.

To standardize color, salmon farms use the Roche SalmoFan™ scale (refer to Supplementary Figure S1), a physical fan with numbered color chips (typically 20–34), to guide feed adjustments based on market preferences. Shades 26–28 are especially favored for their visual appeal. While the SalmoFan™ primarily measures pigmentation (astaxanthin levels), it also indirectly reflects freshness, as color fades with age or improper storage.

Farmed salmon is widely favored over wild salmon due to its consistent availability, lower production costs, and reliable quality. Unlike wild salmon, which is only available during specific seasons, farmed salmon can be harvested year-round, ensuring a stable supply for global markets. Wild salmon and farmed salmon meat differ significantly in terms of their origin, diet, environment, and resulting characteristics, which impact their appearance, flavor, texture, nutritional content, and suitability for various culinary applications. Table 2 contrasts wild and farmed salmon by key visual traits. Wild salmon typically shows a deep red–orange color with natural gradients, thin, sparse fat lines, firmer, denser muscle, a tapered fillet with varied thickness, and a more matte finish. Farmed salmon tends to be uniformly light pink–orange (often feed-influenced), with thick, prominent white marbling, a softer “buttery” texture, a more uniform, thicker fillet, and a glossier, oilier surface due to higher fat. Because feed colorants (e.g., astaxanthin) can deepen farmed color, marbling and texture are more reliable identifiers than hue alone.

### 1.3. Research Motivation and Purpose

Seafood fraud, such as mislabeling rainbow trout as salmon, poses risks to consumer health and undermines trust in the seafood market. Existing laboratory-based methods for species authentication and freshness grading are accurate but costly, time-consuming, and inaccessible to consumers. This study aims to develop a practical, smartphone-compatible system for fish meat classification and salmon freshness grading using deep learning. The goal is to empower consumers to verify authenticity and freshness at the point of purchase or consumption, supporting food safety and consumer rights.

### 1.4. Challenges in Image-Based Fish Meat Classification

Despite its practicality, image-based classification faces challenges due to the visual similarities between salmon and rainbow trout, variability introduced by lighting and camera angles, and subtle differences in marbling and texture. Additionally, limited and imbalanced datasets complicate model training and risk bias. Addressing these challenges requires robust architectures capable of learning fine-grained visual distinctions while maintaining efficiency for deployment on mobile devices. This study tackles these challenges by utilizing an improved DenseNet121 architecture and transfer learning.

This paper begins with a review of current methods for fish meat classification and freshness grading. It then presents a proposed deep learning-based sensing system for identifying salmon meat and grading its freshness. The study continues with performance and robustness evaluations, and concludes by summarizing key contributions and outlining directions for future research.

## 2. Literature Review

Seafood fraud poses serious risks to food safety and consumer rights. Traditional identification methods like manual inspection and chemical analysis often lack real-time applicability for consumers. Recent studies have explored visual, spectroscopic, and machine learning techniques to address these challenges. Among these, deep learning approaches have shown promise for automating fish meat classification and freshness grading. This literature review highlights existing research on seafood fraud detection, fish meat classification methods, freshness evaluation techniques, and deep learning models, establishing a foundation for the proposed system.

### 2.1. Food Fraud and Safety in the Seafood Industry

Seafood fraud presents a widespread and growing concern globally. Studies have shown mislabeling rates to average around 8–20% depending on region and season, with restaurant offerings during the off-season reaching even higher levels [2,4,5]. This type of fraud not only deceives consumers economically but also raises public health concerns; for instance, trout carries a higher risk of parasites and is not suitable for raw consumption. To combat these risks and uphold consumer rights, numerous detection strategies have emerged, with DNA barcoding and SNP analysis providing highly accurate species identification [6,7]. However, such methods are often resource-intensive and not ideal for routine or on-site verification.

In response to these limitations, recent studies have explored more rapid and accessible approaches. Techniques like MALDI-TOF protein fingerprinting and lateral flow-based PCR assays aim to reduce processing time while maintaining reliability [8,9]. In parallel, regulatory bodies are increasingly implementing traceability systems and enforcement actions to deter fraudulent practices. Despite these efforts, consumer awareness remains relatively low, underscoring the need for practical detection tools that empower buyers directly. Therefore, image-based, deep learning solutions, such as smartphone-compatible classification systems, offer a promising alternative, enabling real-time, cost-effective detection of species mislabeling and freshness, ultimately contributing to food safety and the protection of consumer interests.

### 2.2. Fish Meat Classification Methods

Fish meat classification traditionally relied on manual inspection and analytical techniques. Visual inspectors assess fillet color, marbling, and texture, but this method is subjective and inconsistent due to lighting conditions and human variability [10]. Biochemical assays, like fatty acid profiling or spectrophotometric pigment analysis, offer greater objectivity but are destructive, time-consuming, and unsuitable for real-time use in processing lines or at points of sale [11].

With advances in imaging and machine learning, noninvasive, automated classification methods have become more reliable and accessible [12,13]. Spectroscopic methods, such as near-infrared (NIR) spectroscopy and hyperspectral imaging (HSI), capture chemical signatures that differentiate species and origin based on subtle differences in muscle composition [14]. Computer vision and machine learning approaches, utilizing color histograms, texture descriptors, and handcrafted features combined with classifiers like SVM and Random Forest, have shown promising accuracy [15]. More recently, deep learning models (e.g., CNNs like DenseNet, ResNet) have outperformed traditional techniques by automatically extracting discriminative features from fish meat images [16]. Deep learning models like DenseNet and ResNet offer automated feature extraction, and transfer learning enhances performance even with limited training data. These methods represent a significant step toward real-time, consumer-facing inspection systems that are both accurate and cost-effective.

### 2.3. Fish Freshness Evaluation Techniques

Fish freshness has historically been evaluated using sensory assessment and chemical analyses. Sensory methods involve trained panels inspecting fish meat for odor, color, texture, and slime formation, yet these are inherently subjective and prone to inter-observer variability [17]. Objective spoilage indicators include biochemical markers such as total volatile basic nitrogen, trimethylamine, and thiobarbituric acid reactive substances [18,19]. Although accurate, these assays are labor-intensive, destructive, and often require lab equipment, making them unsuitable for rapid, on-site freshness assessment, especially at consumer or retail levels.

To overcome these limitations, researchers have turned toward non-invasive, rapid methods like spectroscopy and computer vision. Techniques such as near-infrared (NIR), hyperspectral imaging (HSI), and Raman spectroscopy enable the detection of chemical changes in fish tissues related to freshness, including moisture, protein, and pigment breakdown [20,21,22,23]. However, these systems are often expensive and require controlled lighting conditions. Meanwhile, image-based approaches using deep learning models, especially convolutional neural networks (CNNs), have emerged as scalable alternatives. By analyzing color, marbling, and texture in RGB images, models like DenseNet, ResNet, and MobileNet can classify freshness levels with high accuracy [24,25]. These approaches offer real-time, non-destructive, and consumer-accessible grading of fish freshness, laying the groundwork for practical tools such as mobile applications and automated retail sensors.

### 2.4. Deep Learning Approaches for Fish Meat Classification and Freshness Grading

Deep learning has revolutionized the field of fish meat classification by enabling models to automatically extract and learn complex image features such as marbling, color gradients, and texture patterns, all critical for distinguishing subtle differences in species (e.g., wild vs. farmed salmon, trout impersonating salmon) and cut types. CNNs like DenseNet, ResNet, and Inception have demonstrated high accuracy in fish-related tasks [26,27]. DenseNet architectures are well-suited for fish classification tasks, offering high accuracy with fewer parameters through dense connectivity, efficient gradient propagation, and feature reuse [28]. Tupan et al. [29] compared DenseNet with ResNet and Inception models for tuna loin classification under various treatment conditions. DenseNet achieved the highest accuracy, confirming its effectiveness in capturing fine-grained visual differences among fish meat types.

Additionally, DenseNet has been used in fish freshness prediction and species recognition. Genç et al. [30] applied DenseNet121 with Gradient-weighted Class Activation Mapping (Grad-CAM) and LIME explainability tools to non-destructively assess seabream freshness, highlighting its suitability for quality assurance in aquaculture. Meanwhile, Malik et al. [31] proposed an improved DenseNet-169-based model to classify underwater fish species with higher accuracy than YOLO and traditional CNN models, demonstrating DenseNet’s robustness in visually complex environments. These findings confirm that DenseNet is a highly suitable backbone for automated fish meat classification and quality grading systems [32].

For freshness grading, deep learning models are increasingly employed to assess visual indicators like flesh color, texture, and fat distribution, offering a scalable, non-destructive alternative to traditional sensory or chemical assays [33]. Models such as CNNs trained on RGB images have demonstrated the ability to classify freshness levels effectively, with transfer learning providing the additional benefit of improved accuracy under varied imaging conditions [24]. These methods collectively highlight the promise of deep learning in delivering rapid, accessible, and objective tools for both fish meat authentication and freshness assessment.

Despite significant advancements in fish meat classification and freshness assessment using deep learning techniques, a critical gap remains in developing integrated systems that can simultaneously perform species identification, evaluate growth and cutting types, and grade freshness levels in a user-friendly and real-time manner. Most existing studies focus on either species classification [34,35] or freshness grading [24,25,33] separately, often relying on laboratory conditions or requiring specialized imaging equipment. Similarly, image-based deep learning studies have explored fish species classification in controlled environments, but few have addressed the practical deployment of such methods in everyday consumer contexts. To bridge this gap, this study introduces a smartphone-compatible, deep learning–based classification system that identifies salmon and rainbow trout meat and grades salmon freshness. By utilizing an improved DenseNet121 architecture and transfer learning, the proposed system offers both high accuracy and computational efficiency, making it practical for mobile applications. Unlike prior methods, this approach emphasizes accessibility, cost-effectiveness, and consumer usability, empowering end-users to verify seafood authenticity and freshness in real-world purchasing environments.

## 3. Materials and Methods

The research workflow of this study consists of four parts. (1) Image acquisition: fish-meat images are gathered from web sources and from our own photography, ensuring complete coverage of each category, and then cropped to appropriate sizes. (2) Network model building and training: two tasks are addressed, fish meat classification and salmon freshness grading, both trained and predicted using the DenseNet121 architecture. (3) Performance evaluation: the effectiveness and efficiency of classification and grading are assessed using recall, precision, accuracy, and training time. (4) Method comparison: evaluating DenseNet121 against five other commonly used deep learning models to identify the most suitable approach for fish-meat classification and freshness grading.

### 3.1. Data Acquisition and Smartphone-Based Image Capture

In this study, three distinct salmon cuts, steak, fillet, and sashimi, are used for both classification and freshness grading tasks. A salmon steak is a cross-sectional cut taken perpendicular to the spine, typically 1–2 inches thick, often including the central bone and skin, and exhibiting pronounced white fat marbling within vibrant orange-red flesh. A fillet is a boneless, lengthwise cut parallel to the spine, producing a tapered, flat piece (approximately 0.5–1 inch thick) with similar coloration but without the central bone. Sashimi refers to thin, skinless, boneless slices (about 0.125–0.25 inches thick) cut from sushi-grade fillets for raw consumption, characterized by their glossy and uniform flesh color. These cuts are selected because they represent the most common consumer presentations of salmon and provide distinct visual characteristics, color, marbling, and texture that are critical for both species differentiation and freshness assessment. Each cut type was carefully imaged and categorized to build a robust dataset for training and testing the proposed deep learning models.

**Figure 1:**
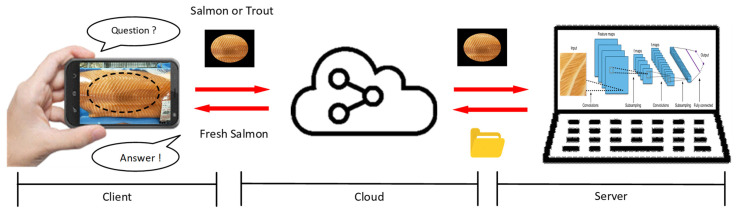
System architecture for image-based fish meat identification and freshness grading using cloud-based deep learning inference.

This study adopts a vision-based sensing approach, where customers using smartphones capture RGB images of fish meat that are then processed by deep learning algorithms for classification and grading. Figure 1 illustrates a cloud-based deep learning classification system for fish meat identification and freshness grading. It consists of three main components: the client, cloud, and server. On the client side, users capture an image of the fish meat using a mobile device and query whether it is salmon or trout. This image is transmitted via the cloud to a server equipped with a deep learning model. The server processes the image using a CNN to identify the fish type and evaluate its freshness level. The classification result—such as “Fresh Salmon”—is then sent back to the client through the cloud, allowing consumers to make informed decisions. This setup enables real-time image-based fish product verification and freshness assessment through mobile applications.

### 3.2. Image Pre-Processing and Segmentation

A single captured image of a salmon steak is segmented into six distinct grid regions, each highlighting different parts of the meat. These cropped grid images are then used to build the dataset, with a portion allocated for training the model and the remainder reserved for testing its performance. This grid-based approach enhances the model’s ability to learn diverse texture and marbling features from various meat sections, thereby improving classification accuracy for both training and evaluation phases.

**Figure 2:**
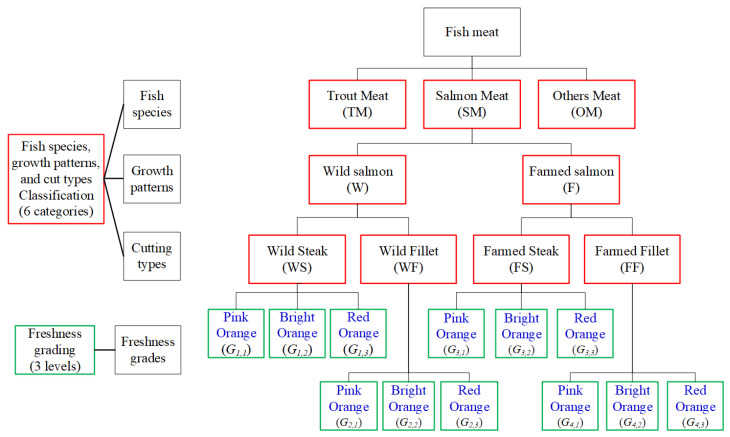
Classification tree diagram of fish meat categorization and freshness grading.

Figure 2 presents a hierarchical classification framework for fish meat, integrating species, growth patterns, cutting types, and freshness grading. At the top level, fish meat is categorized into three primary species groups: trout meat (TM), salmon meat (SM), and other meat (OM). Salmon meat is further divided by growth patterns into wild salmon (W) and farmed salmon (F). Each growth type is then classified by cut type into steak and fillet categories, resulting in four subgroups: wild steak (WS), wild fillet (WF), farmed steak (FS), and farmed fillet (FF). Each of these six fish meat categories undergoes freshness grading, which is stratified into three levels based on color indicators: pink orange (high quality), bright orange (premium quality), and red orange (low quality). These freshness grades of salmon meat are represented as $G_{i,\;j}$, where *i* denotes the category of combining growth patterns and cutting types, and *j* the freshness level. This structured model supports fine-grained classification and grading of fish meat for quality assessment and consumer guidance.

When training and predicting salmon freshness using deep learning models, each image must first be manually labeled with its corresponding freshness level. While standard color reference scales in literature include 15 distinct levels, this study simplifies the classification into 3 levels. These three reference colors are visually compared against each image sample, and, based on this comparison, a ground-truth freshness label is assigned manually to each image, completing the annotation process for model training.

**Figure 3:**
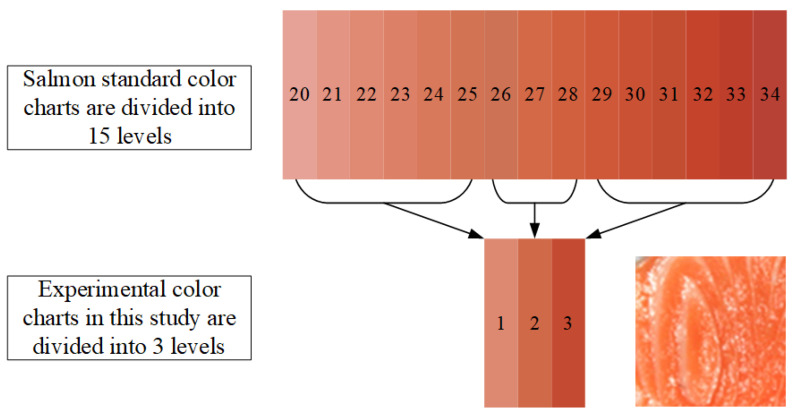
Salmon standard color chart and the corresponding 3-level experimental color chart in this study.

Figure 3 presents a visual comparison between the standard salmon color chart and the simplified 3-level experimental color chart employed in this study. The top portion of the figure displays the Roche SalmoFan™ standard, which classifies salmon flesh color into 15 distinct levels, ranging from level 20 (light pink) to level 34 (deep red-orange). To streamline analysis and enhance model performance, these 15 levels were grouped into three broader categories for experimental use. As illustrated in the middle of the figure, color levels 20 to 24 were classified as Level 1, representing Pink Orange; levels 25 to 28 as Level 2, denoting Bright Orange; and levels 29 to 34 as Level 3, indicating Red Orange. Level 2 bright orange is more favored by consumers due to its fresher appearance, which results from a higher astaxanthin content. However, the color of fish meat is not necessarily better the redder or the whiter it is. This reclassification allowed for more efficient mapping of salmon images (as shown in the lower right corner) to a simplified color grade structure while preserving the essential distinctions relevant to freshness evaluation and consumer perception.

To ensure the three-level system’s validity and practicality, we map the levels to ranges of the 15-level SalmoFan™ chart based on perceptual similarity and expert judgment. Seafood specialists and quality inspectors then annotate images against physical SalmoFan™ cards, and consensus labels are used to confirm consistent color categorization. Quantitative testing shows high accuracy with minimal class confusion, indicating the grouped labels retain sufficient discriminative power for freshness assessment. While finer gradations may suit premium supply chains, a three-level scheme offers a clear, reliable, and user-friendly balance for consumer protection and fraud detection.

### 3.3. Deep Learning Models for Salmon and Rainbow Trout Classification

DenseNet (Densely Connected Convolutional Network) [28] is renowned for its densely connected architecture, which facilitates efficient feature reuse and gradient flow, enabling the model to effectively learn both low-level textures and high-level semantics essential for distinguishing subtle variations in marbling, fat distribution, and color in fish meat. Its compact design requires fewer parameters compared to other deep networks, reducing the risk of overfitting—an advantage when working with relatively small datasets often found in food image analysis. Additionally, DenseNet excels in transfer learning scenarios, allowing pretrained models to adapt well to new tasks with minimal fine-tuning, which is beneficial for handling real-world variations in fish presentation, such as lighting, packaging, or slicing differences.

#### 3.3.1. DenseNet121 Model

DenseNet121, introduced by Huang et al. (2017), is a convolutional neural network characterized by dense connectivity, where each layer receives inputs from all preceding layers [28]. This architecture promotes feature reuse, efficient gradient flow, and reduces the vanishing gradient problem, making it well-suited for image classification tasks with limited computational resources.

**Figure 4:**
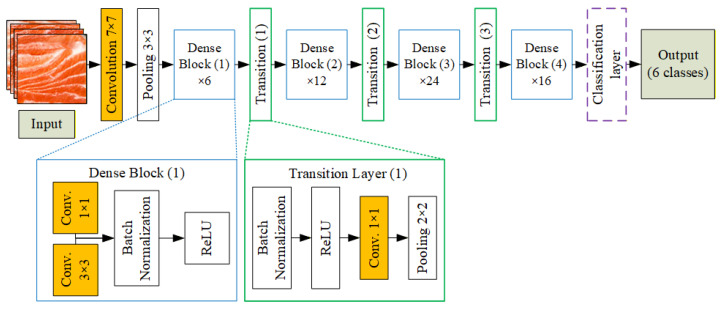
Architecture of the DenseNet121-based fish meat classification model.

Figure 4 shows the DenseNet121 architecture applied in this study. Input images of salmon meat are first processed through a 7 × 7 convolution layer, followed by 3 × 3 max pooling. The core of the network consists of four dense blocks (with 6, 12, 24, and 16 layers, respectively), each containing 1 × 1 and 3 × 3 convolutions. Between the dense blocks are three transition layers for downsampling, each composed of batch normalization, ReLU, a 1 × 1 convolution, and 2 × 2 average pooling. The final feature maps are passed to a classification layer. After feature extraction, a fully connected dense layer with 128 ReLU-activated neurons processes the feature vector. A dropout layer (rate = 0.2) helps prevent overfitting. Finally, a softmax layer outputs probabilities across six classes: wild steak, wild fillet, farmed steak, farmed fillet, trout meat, and other meat types. This structure enables accurate fish meat classification based on visual characteristics like color, marbling, and texture.

#### 3.3.2. Improved DenseNet121 with Global Average Pooling

To improve classification performance and enhance training efficiency, the original DenseNet121 architecture is modified using transfer learning techniques. This process involved fine-tuning selected layers, freezing others to retain learned features, and structurally adapting the network to better suit the specific task of fish meat classification and salmon freshness grading. The modified architecture, termed Improved DenseNet121, incorporates four key enhancements: a Global Average Pooling (GAP) layer, a dropout layer, a fully connected layer, and a final output layer [36,37].

The GAP layer replaces traditional flattening by averaging each feature map, transforming multi-dimensional outputs into a compact one-dimensional vector. This design drastically reduces trainable parameters compared to fully connected layers, lowering overfitting risk and improving generalization, particularly for small or specialized datasets like fish meat classification. GAP also preserves spatial context, enhances interpretability, and improves computational efficiency, making it a preferred choice in modern CNNs. To further strengthen robustness, dropout layers randomly deactivate neurons during training, while a fully connected layer maps extracted features to class scores, and a softmax layer generates final classification probabilities. Together, these enhancements enable DenseNet121 to capture key visual features effectively while maintaining both accuracy and computational efficiency.

**Figure 5:**
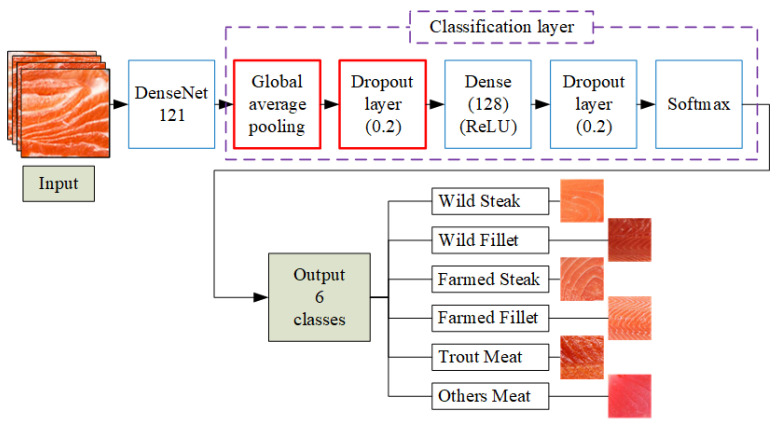
Architecture of the improved DenseNet121-based fish meat classification model.

Figure 5 illustrates the architecture of the improved DenseNet121 model for fish meat classification. Input images are first processed by the DenseNet121 backbone to extract deep features. These features are then passed through a GAP layer to reduce spatial dimensions and computational load. A dropout layer (0.2) is applied to mitigate overfitting, followed by a fully connected dense layer with 128 ReLU-activated neurons. Another dropout layer is added before the final softmax layer, which outputs probabilities across six meat categories. This enhanced structure improves the model’s generalization and classification accuracy, especially for visually similar classes.

### 3.4. Transfer Learning of Source Domains and Weights

**Figure 6:**
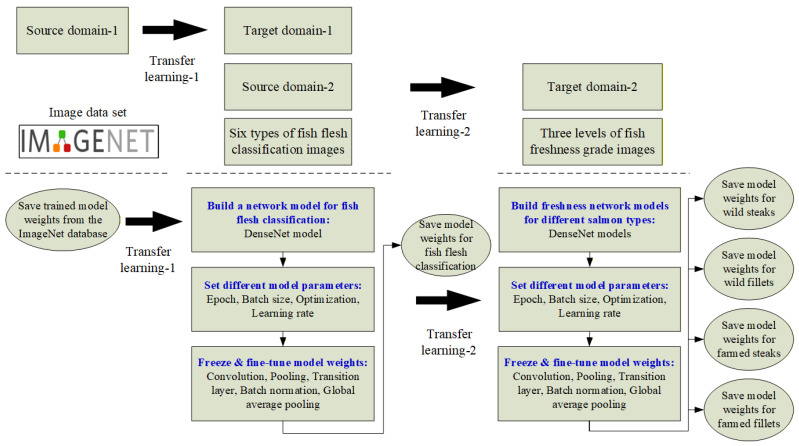
Schematic diagram of transfer learning for fish meat classification and freshness grading.

During the training phase of a deep learning model, each neuron requires an initial weight (W), which can either be randomly initialized or transferred from a previously trained model. The use of pre-trained weights for training a new model is known as transfer learning (TL). As illustrated in Figure 6, this study adopts a two-stage transfer learning approach. In the first stage, pre-trained weights from the ImageNet database are used as the source domain, while the target domain consists of fish meat images. In the second stage, the pre-trained weights obtained from the fish meat classification model in the first stage are reused as the source domain for the salmon freshness grading task, resulting in improved classification performance in the freshness grading process.

A common method for implementing transfer learning is to freeze the lower layers of a pre-trained model—typically the early convolutional layers—while fine-tuning the later layers with new data. This involves keeping the feature extraction layers unchanged and retraining only the upper layers, especially those closer to the output. The final classification layer is modified to match the number of categories in the new dataset. During fine-tuning, a smaller learning rate is applied to avoid overwriting learned features. If the dataset is small, only the last layer is trained; for medium-sized datasets, early layers are frozen while later layers are updated.

The classification and grading methods in this study are implemented using transfer learning, with the Improved DenseNet121 model serving as the adapted network. To optimize performance, experiments are conducted by freezing different portions of the model’s 426 layers, targeting either low-level or high-level feature extractors. Eleven configurations are tested, with layer freezing applied in 10% increments from 0% to 100%, as detailed in Table 3. Each configuration included three components: frozen layers (blue), fine-tuned layers (green), and modified classification layers (red). The final classification layers are adjusted in every case to match the new task. These experiments aim to identify the configuration that provides the best trade-off between accuracy and efficiency while preserving valuable pre-trained features.

The integration of DenseNet121 with transfer learning in this study follows three key steps. First, data augmentation techniques, including scaling, translation, and flipping, are applied to expand the dataset and increase feature diversity, thereby enhancing model robustness. Second, DenseNet121 serves as the core deep learning backbone, consistently applied across all experiments to extract hierarchical features. Finally, the classification stage incorporates GAP and dropout layers, which simplify feature maps, reduce overfitting, and generate reliable class predictions.

### 3.5. Integrated System for Salmon Meat Classification and Freshness Grading

**Figure 7:**
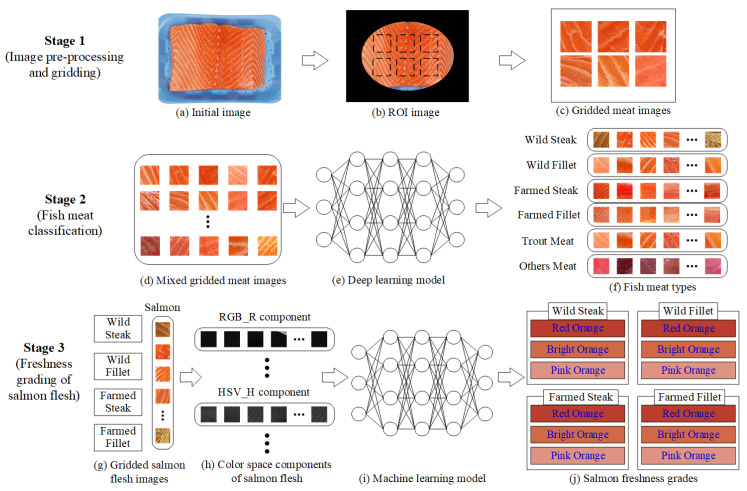
Stage diagram of the salmon classification and grading system.

The developed system is an end-to-end system (smartphone capture, cloud processing, classifier output), emphasizing consumer accessibility, real-time results, and integration of classification and grading models. Figure 7 presents a three-stage framework for automated fish meat classification and salmon freshness grading using image analysis and machine learning. In Stage 1, the process begins with an initial fish image (a), from which a region of interest (ROI) is extracted (b) and divided into smaller gridded meat patches (c). In Stage 2, these mixed gridded images (d) are input into a deep learning model (e) to classify them into six fish meat types (f): wild steak, wild fillet, farmed steak, farmed fillet, trout meat, and other meat types. Stage 3 focuses on assessing the freshness of classified salmon images (g) by analyzing their RGB and HSV color space components (h), which are then processed by a machine learning model (i) to assign freshness grades (j) across three levels: pink orange, bright orange, and red orange. This system integrates classification and grading to enhance the accuracy and efficiency of fish product evaluation.

In steps (b) and (c) of Stage 1, each grid patch inherits the label of its source fillet to enable large-scale, consistent training without the prohibitive effort of expert patch-level annotation. To minimize label–patch mismatch, we (1) select central ROIs with uniform flesh before grid division to avoid edges and background, (2) apply patch quality filtering to remove crops with artifacts, skin, shadows, or glare, (3) calibrate grid size so patches are large enough to capture marbling/texture yet small enough for effective augmentation, and (4) validate empirically, finding minimal accuracy differences between central-only and full-patch training, which supports the fidelity of the inherited-label strategy.

In our pipeline, patches inherit the parent fillet’s label (species/cut or freshness), but we reduce label–patch mismatches in three ways. First, ROIs are restricted to flesh-only regions: we generate a binary flesh mask and discard any grid patch with <80% flesh pixels or contamination by skin, bloodline, glare, or background; edge-adjacent patches are excluded a priori. Second, we use context-preserving patches (224 × 224 with 50% overlap) to retain sufficient marbling/texture cues so a small crop remains representative of the fillet-level label; illumination normalization (white balance/color constancy) reduces local color distortion. Third, at inference, we aggregate patch predictions (majority voting) to form the image-level decision, limiting the impact of any residual ambiguous patches.

In step (i) of Stage 3, in addition to deep learning models, several classical machine learning approaches are tested for salmon freshness grading. Backpropagation Neural Network (BPN) is a feed-forward neural network trained with backpropagation, using RGB and HSV color features as input to classify fish meat [38]. The Fuzzy Inference System (FIS) applies fuzzy rules and membership functions to color inputs, capturing uncertainty but with limited performance on complex image data [39]. The Adaptive Neuro-Fuzzy Inference System (ANFIS) integrates neural networks and fuzzy logic to learn fuzzy rules automatically, offering nonlinear adaptability with rule-based interpretability [40]. These baseline methods, relying on hand-crafted color features, provided useful benchmarks, but the proposed Improved DenseNet121, trained directly on RGB images, achieved superior performance by utilizing richer image-level features.

Although color cues (RGB/HSV components) form the most direct indicator for freshness, the improved DenseNet121 model also captures additional visual features such as texture, marbling distribution, structural patterns, and surface gloss through its convolutional layers. By learning multi-scale features from raw images, the network integrates both color and non-color cues, enabling it to identify freshness-related differences that extend beyond simple pigmentation levels.

### 3.6. Combined Categorization of Fish Classification and Freshness Grading

The classification problem in this study follows a two-stage approach. If fish meat classification and freshness grading are combined into a single step, a comparison between the two-stage and one-stage methods is further investigated. In the two-stage approach, fish meat is first classified into six categories, followed by freshness grading applied only to four salmon categories. Each of these four categories is further subdivided into three freshness levels, yielding a total of 6 (fish types) + 12 (freshness grades) = 18 distinct classes. Conversely, the one-stage approach attempts to classify the input directly into 14 categories: 12 combinations of salmon type and freshness, along with two additional classes for other fish types.

This comparative setup enables a performance evaluation between modular (two-stage) and integrated (one-stage) classification strategies. While the two-stage method potentially allows for specialized fine-tuning in each phase, the one-stage model benefits from end-to-end optimization. Both models are trained and evaluated on the same dataset to ensure fairness and consistency. Their performance is analyzed using standard metrics such as accuracy, F1-score, and confusion matrices, as discussed in Section 4.

## 4. Results and Discussion

To validate the proposed system for identifying fish species, growth environments, cutting types, and grading salmon freshness, this study conducts practical experiments and performance evaluations. Results are benchmarked against alternative methods to assess effectiveness, followed by sensitivity analyses to explore additional influencing factors.

### 4.1. Experimental Hardware, Captured Images, and User Interface

This study utilized the following hardware configuration: a personal computer equipped with an Intel^®^ Core™ i7-10700F CPU, 32GB RAM, and an NVIDIA GeForce RTX™ 3070 GPU, running Windows 10 Professional. The software environment included Python 3.8.1, TensorFlow 2.7.3, and MATLAB R2021b. The experimental images used in this study are fish meat images, each resized to 224 × 224 pixels. Table 4 presents the distribution characteristics and descriptions of fish meat and fat across different image categories.

The user interface of the automated fish meat classification and salmon freshness grading system is illustrated in Supplementary Figure S2.

### 4.2. Training and Testing Sample Numbers of Fish Meat Category and Freshness Grade

In the first stage of fish meat classification, the samples are divided into six categories: Wild Steak (WS), Wild Fillet (WF), Farmed Steak (FS), Farmed Fillet (FF), Trout Meat (TM), and Other Meat (OM). The number of training and testing images for each category is 160 and 80. In the second stage, each of the four salmon categories undergoes freshness grading, classified into three levels: Pink Orange (PO), Bright Orange (BO), and Red Orange (RO). The training and testing image counts for each freshness category are 120 and 60. The detailed composition of the training and testing datasets used for fish meat classification and salmon freshness grading is provided in Supplementary Tables S1 and S2.

### 4.3. Network Model Selection and Parameter Settings

In the fish meat classification stage, DenseNet121 is selected as the base model, with input image dimensions set to 224 × 224 pixels. The training parameters are configured with a learning rate of 0.0001, batch size of 8, and 80 training epochs. Following this, various combinations of DenseNet versions, learning rates, batch sizes, training epochs, and other parameters are compared to determine the most effective configuration for optimal classification performance.

### 4.4. Comparison of Detection Effectiveness of Different Models

This study visualizes the model’s classification results using confusion matrices, followed by the calculation of performance evaluation metrics, including precision, recall, F1-score, and classification accuracy. To thoroughly assess classification performance, the analysis focuses on the classification outcomes of each individual category.

#### 4.4.1. Performance Evaluation Indices and Confusion Matrix of Experimental Results

In the first and second stages, the quantities of classification results are denoted by *F* and *S*, representing fish meat classification and salmon freshness grading, respectively. To evaluate the performance of the proposed fish meat identification and salmon freshness grading system, this study presents two confusion matrices, summarized in Table 5 and Table 6, for fish meat classification and freshness grading, respectively. Standard classification metrics are used for evaluation, including recall, precision, accuracy, and F1-score, where the F1-score represents the harmonic mean of precision and recall. Higher F1-score and accuracy values indicate stronger overall detection performance and greater model reliability. In both confusion matrices, the classification metrics are illustrated using the first category as an example; the similar definitions and calculation methods apply to the remaining categories.

For fish meat identification, the recall rate or detection rate for wild steak (WS) of salmon is defined as:

(1) $$Recall\text{\_}WS(\%)=\left(\frac{Number\;of\;images\;correctly\;classified\;as\;wild\;steak\;of\;salmon}{Total\;number\;of\;images\;for\;wild\;steak\;of\;salmon}\right)\times 100\%=\frac{F_{1,1}}{F_{1,\cdot }}\times 100\%$$

Precision rate for wild steak of salmon is defined as:

(2) $$Precision\text{\_}WS(\%)=\left(\frac{Number\;of\;images\;correctly\;classified\;as\;wild\;steak\;of\;salmon}{Total\;number\;of\;images\;detected\;as\;wild\;steak\;of\;salmon}\right)\times 100\%=\frac{F_{1,1}}{F_{\cdot ,1}}\times 100\%$$

F1-Score rate for wild steak of salmon is defined as:

(3) $$F1-score\text{\_}WS(\%)=\left(\frac{2\;\times \;Recall\text{\_}WS\;\times \;Precision\text{\_}WS}{Recall\text{\_}WS\;+\;Precision\text{\_}WS}\right)\times 100\%$$

Accuracy rate for fish meat (FM) classification is defined as:

(4) $$Accuracy\text{\_}FM(\%)=\left(\frac{Number\;of\;correctly\;classified\;images}{Total\;number\;of\;test\;images}\right)\times 100\%=\frac{\sum_{i=1}^{6}F_{i,i}}{F_{\cdot ,\cdot }}\times 100\%$$

For salmon freshness grading, the recall rate or detection rate for the pink-orange (PO) level of salmon freshness is defined as:

(5) $$Recall\text{\_}PO(\%)=\left(\frac{Number\;of\;images\;correctly\;classified\;as\;pink-orange\;level\;of\;salmon\;freshness}{Total\;number\;of\;images\;for\;pink-orange\;level\;of\;salmon\;freshness}\right)\times 100\%=\frac{S_{1,1}}{S_{1,\cdot }}\times 100\%$$

Precision rate for the pink-orange level of salmon freshness is defined as:

(6) $$Precision\text{\_}PO(\%)=\left(\frac{Number\;of\;images\;correctly\;classified\;as\;pink-orange\;level\;of\;salmon\;freshness}{Total\;number\;of\;images\;detected\;as\;pink-orange\;level\;of\;salmon\;freshness}\right)\times 100\%=\frac{S_{1,1}}{S_{\cdot ,1}}\times 100\%$$

F1-Score rate for the pink-orange level of salmon freshness is defined as:

(7) $$F1-score\text{\_}PO(\%)=\left(\frac{2\;\times \;Recall\text{\_}PO\;\times \;Precision\text{\_}PO}{Recall\text{\_}PO\;+\;Precision\text{\_}PO}\right)\times 100\%$$

Accuracy rate for salmon freshness (SF) grading is defined as:

(8) $$Accuracy\text{\_}SF(\%)=\left(\frac{Number\;of\;correctly\;classified\;images}{Total\;number\;of\;test\;images}\right)\times 100\%=\frac{\sum_{i=1}^{3}S_{i,i}}{S_{\cdot ,\cdot }}\times 100\%$$

In terms of efficiency indicators, it is important to distinguish between training time and inference time in this study. Training time refers to the total computational effort required to optimize the model parameters on batches of training images at the server side; this metric is mainly used for comparing the efficiency of different architectures and training strategies. In contrast, inference time refers to the time needed to process and classify a single test image once the model has been trained, which directly determines the feasibility of real-time deployment on consumer devices. In our experiments, the improved DenseNet121 achieved an average inference time of approximately 45–60 milliseconds per image on a standard GPU, corresponding to near real-time performance for smartphone-based applications. While training time is relevant for research and efficiency analysis, inference time is the critical metric for end-users, as it reflects how quickly the system can deliver classification and freshness grading results in practice.

#### 4.4.2. Classification Results of Fish Meat

**Figure 8:**
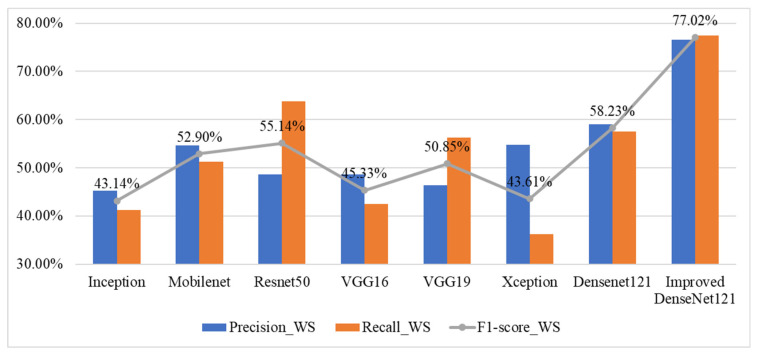
Performance indicators for wild salmon steak classification across various deep learning models.

Figure 8 presents a comparative analysis of performance indicators: precision, recall, and F1-score, for wild salmon steak classification using various deep learning models. Among the eight models evaluated, the Improved DenseNet121 demonstrates the highest performance across all metrics, achieving 77.02% in F1-score. In contrast, models like Inception and Xception show relatively low performance, with F1-scores below 45%. DenseNet121 and ResNet50 also perform moderately well, with F1-scores around 58.23% and 55.14%, respectively. Overall, the results highlight that enhancing the DenseNet121 architecture significantly boosts classification accuracy for wild salmon steak images.

**Figure 9:**
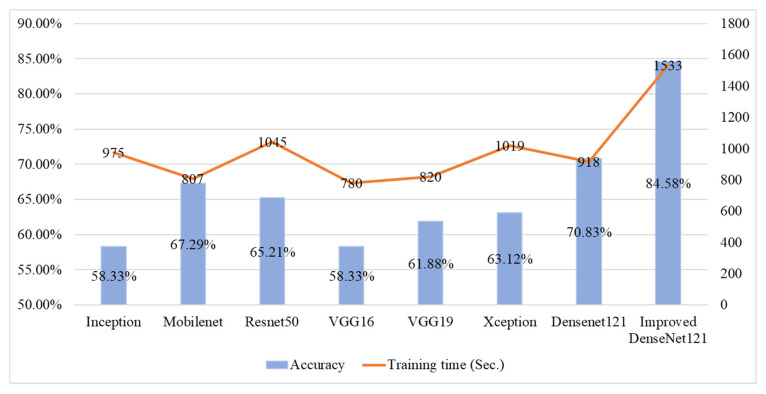
Comparative results of accuracy and training time across different deep learning models for fish meat classification.

Figure 9 presents a comparison of the average classification accuracy and training time for various deep learning models applied to fish meat classification. The Improved DenseNet121 model achieves the highest accuracy at 84.58%, but it also incurs the longest training time of 1533 s, reflecting a trade-off between accuracy and computational efficiency. In contrast, Mobilenet shows the shortest training time (807 s) while maintaining a moderate accuracy of 67.29%, making it more suitable for resource-constrained scenarios. Other models like ResNet50 and DenseNet121 offer a balance, with ResNet50 achieving 65.21% accuracy in 1045 s, and DenseNet121 reaching 70.83% in 918 s. Overall, this figure highlights that while deeper or improved architectures enhance accuracy, they also increase training time significantly.

To ensure fair comparisons, all models were trained and tested on the same dataset of fish meat and freshness images. This approach guarantees that performance differences reflect the architectures and configurations rather than dataset variations. While this strengthens internal validity, it also introduces certain limitations. Using a single dataset may inadvertently bias model learning toward specific image characteristics, such as lighting, background uniformity, or acquisition conditions. Such dataset dependence could limit generalizability when applied to broader real-world environments.

#### 4.4.3. Experimental Results of Adding Transfer Learning

The experimental results of adding transfer learning are summarized in Table 7, which compares the performance and operational differences between three DenseNet121 models and the Improved DenseNet121 model. The Improved DenseNet121 consistently outperformed the baseline DenseNet121 configurations, achieving the highest accuracy (84.58%). These results highlight the benefits of incorporating global average pooling and dropout layers, as well as the efficiency gains from partial layer freezing. These findings confirm that architectural enhancements and layer-freezing strategies yield both practical efficiency and accuracy gains, emphasizing the robustness of the Improved DenseNet121 for fish meat classification and freshness grading.

Figure 9 compares seven candidate models from the selection stage with the Improved DenseNet121 used in this study in terms of effectiveness and efficiency. In terms of effectiveness, the proposed model attains a higher classification accuracy of 84.58%. In efficiency, its training time is 1533 s, which is 67% longer than the original DenseNet121. When choosing the model, effectiveness is prioritized first, followed by efficiency.

**Figure 10:**
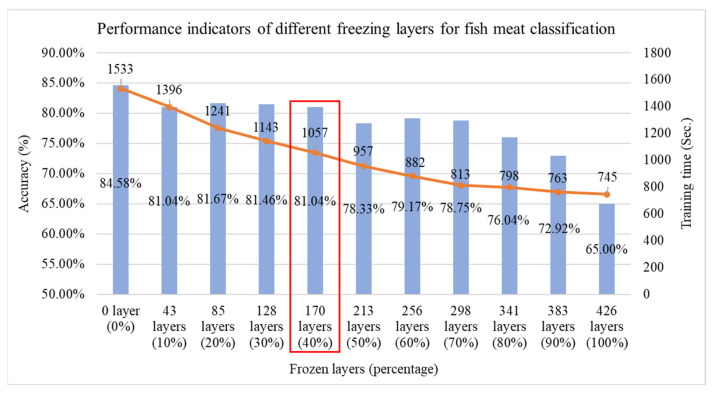
Performance comparison of fish classification using the Improved DenseNet121 model at different freezing layer percentages, red box indicating the optimal selection.

If efficiency is suboptimal, it can be compensated for through other techniques, such as layer freezing in transfer learning. In this study, the model contains a total of 426 layers, and experiments are conducted by freezing layers at 10% intervals from 0% to 100%, as illustrated in Figure 10. As the number of frozen layers increases, training time decreases accordingly. We freeze 40% of DenseNet121’s layers based on a sensitivity study (freeze ratios at 10–50%) to balance efficiency and accuracy. At 40%, training time dropped by ~30% with only a small accuracy decrease (84.58% → 81.04%). Freezing less (e.g., 30%) offered marginal speed gains, while freezing more (e.g., 50%) caused >5% accuracy loss. Thus, 40% provided the optimal trade-off for faster training with minimal performance impact. To balance both effectiveness and efficiency, the model with 40% of its layers frozen is selected, and its trained weights are saved for use in the second-stage salmon freshness grading as the source domain for transfer learning.

Table 8 presents a performance comparison of classification models in the first stage of fish meat classification. The Improved DenseNet121 model shows a 19.41% increase in classification effectiveness compared to the original DenseNet121 but suffers a 66.99% decrease in efficiency (longer training time). When applying transfer learning with 40% of the layers frozen to the Improved DenseNet121 model, effectiveness decreases slightly by 4.18%, but training efficiency improves by 31.05%. To assess whether the accuracy difference (84.58% vs. 81.04%) is statistically meaningful, we apply McNemar’s test on paired predictions from both models over the same test set. The resulting *p*-value was >0.05, indicating no significant difference at the 95% confidence level. Therefore, the small accuracy drop when freezing layers is not statistically significant, supporting the feasibility of this efficiency-focused trade-off. Compared to the original DenseNet121, this transfer learning-enhanced model still achieves a 14.41% gain in effectiveness with only a 15.14% drop in efficiency. Considering the trade-off between performance and computational cost, the study concludes that Improved DenseNet121 with 40% of layers frozen through transfer learning is the optimal model for the first-stage fish meat classification task.

#### 4.4.4. Grading Results of Salmon Freshness

**Figure 11:**
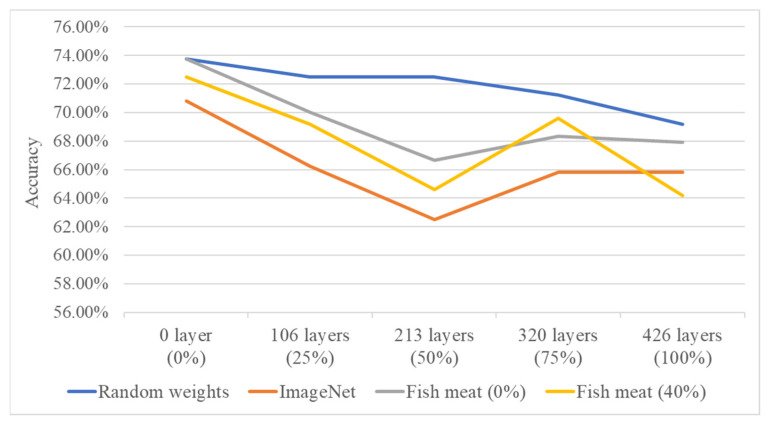
Accuracy comparison of four source domain configurations under varying freezing ratios in the second-stage freshness grading model.

**Figure 12:**
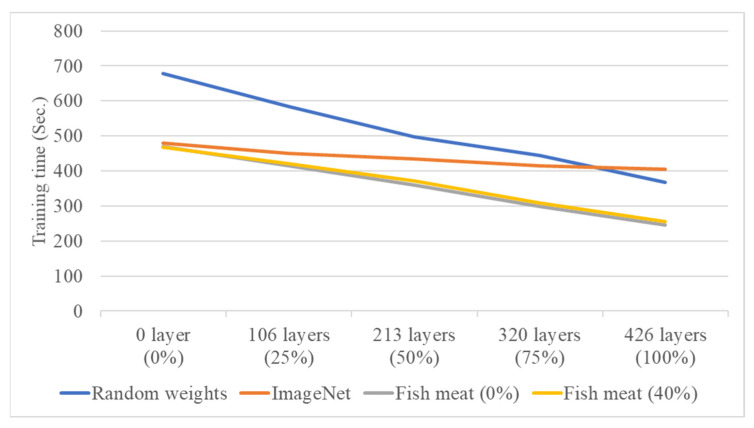
Training time comparison of four source domain configurations under varying freezing ratios in the second-stage freshness grading model.

In the second stage, the system performs individual freshness grading for four types of salmon based on different cutting methods and growth environments. The DenseNet121 model, which showed better performance in the first-stage fish meat classification, is used as the network architecture. After tuning various parameters, the results are visualized using confusion matrices, and classification accuracy and training time are calculated. This study also conducts experiments on transfer learning using four different source domains: randomly initialized weights, pre-trained weights from the ImageNet dataset, pre-trained weights from the first-stage fish meat classification model with 0% layer freezing, and pre-trained weights from the first-stage model with 40% layer freezing. Additionally, different freezing ratios (0%, 25%, 50%, 75%, and 100%) are applied in the second-stage freshness grading model to compare the classification results for the four salmon types. Performance is evaluated based on average classification accuracy (effectiveness) and average training time (efficiency). Figure 11 and Figure 12 show the line charts comparing the effectiveness and efficiency of the four source domain configurations. The results indicate that both the random weight configuration and the 0% freeze configuration from the fish meat classification model yield a higher average accuracy of 73.75%. However, the training time differs significantly—679 s for the random weights and 470 s for the 0% freeze configuration. Therefore, the 0% freeze configuration from the fish meat classification model is selected as the optimal source domain, offering a balance of both accuracy and training efficiency.

Table 9 presents a performance comparison of different source domain weights used in the second-stage salmon freshness grading. Using ImageNet pre-trained weights resulted in 3.96% lower accuracy but 29.31% faster training compared to randomly initialized weights. When switching to weights from the first-stage fish meat classification model with 0% frozen layers, both effectiveness and efficiency improved by 4.12% and 2.08%, respectively, over ImageNet weights. However, using 40% frozen weights from the same model slightly reduced effectiveness by 1.69% while improving efficiency by only 0.43%. Considering both accuracy and training time, the study identifies the 0% frozen fish meat model weights as the optimal source domain for transfer learning in the second-stage salmon freshness grading.

In this study, alongside deep learning models, classical machine learning approaches such as Backpropagation Neural Network (BPN), Fuzzy Inference System (FIS), and Adaptive Neuro-Fuzzy Inference System (ANFIS) are also employed to evaluate salmon freshness. Unlike deep learning models that directly process RGB image data, these machine learning methods require input in the form of feature vectors, which are constructed using both RGB and HSV color components. This design enables a fair benchmark comparison against the image-based deep learning framework. The results of this comparison are presented in Table 10, which shows that the Improved DenseNet121 model with 0% freezing achieved superior accuracy, demonstrating higher effectiveness than the classical machine learning methods.

### 4.5. Comparative Evaluation of One-Stage vs. Two-Stage Classification Approaches

To evaluate classification pipeline design, this study compares the performance of the proposed two-stage approach with a one-stage approach that combines both tasks into a single multi-class problem. Table 11 summarizes the sample distribution and performance outcomes. To control for sample imbalance, particularly the dominance of visually distinct non-salmon fish types, all classes are balanced prior to training and evaluation. The improved DenseNet121 model, enhanced with GAP and regularization layers, consistently outperformed the original version across all scenarios. Specifically, it achieved 84.58% accuracy in the fish meat classification stage and 73.75% in freshness grading under the two-stage setup, compared to 66.50% in the one-stage configuration. The observed drop in accuracy for the one-stage model is primarily due to the increased category complexity, the inherent challenge of learning multiple feature domains simultaneously, and the error propagation reduction in the two-stage setup. The two-stage approach proves more effective by decomposing the task into simpler, sequentially optimized sub-tasks, thus offering better performance and interpretability.

### 4.6. Robustness Analysis of Proposed Approach

To evaluate the robustness of the proposed methods, a sensitivity analysis is conducted on 480 test images (80 per category). The analysis examined the effects of motion blur and camera angle variations on classification accuracy, providing insights into the stability of the system under practical image-capture conditions.

#### 4.6.1. Impact of Object Movement on Classification Effectiveness

**Figure 13:**
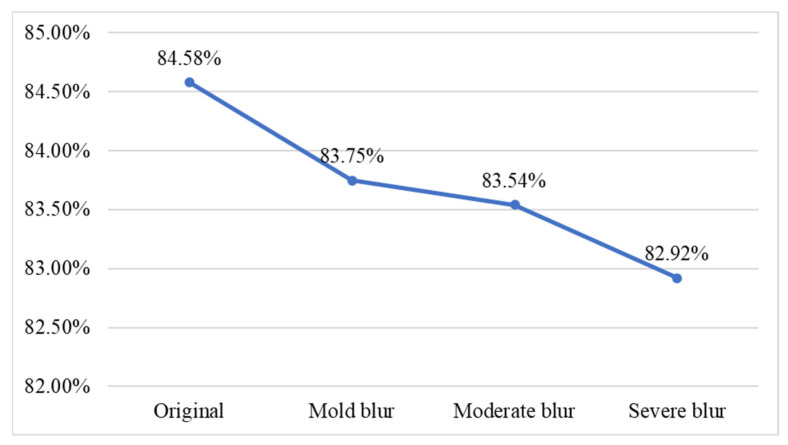
Classification accuracy at different blur levels.

When consumers capture images in real-world environments such as conveyor belt sushi restaurants or supermarkets, image blurring can occur due to object movement. Blurred images may reduce classification accuracy. To investigate this factor, this study applied average filtering to simulate motion blur on fish meat images, replicating the visual effects caused by photographing moving fish. Furthermore, the study examined the tolerance of the proposed network model in classifying blurred images of moving fish. The average filtering method employed three mask sizes to represent different levels of blur: a 3 × 3 mask for mild blur, a 5 × 5 mask for moderate blur, and a 7 × 7 mask for severe blur. Sample images for each blur level are shown in Table 12. We conduct experimental classification on fish meat images with three different levels of blur, and the results are shown in Figure 13. The line chart indicates that, as blur increases, the correct classification rate gradually decreases; however, overall performance remains above 80%, with no significant difference from the results using the original (unblurred) images.

#### 4.6.2. Impact of Tilted Capture Direction and Angle on Classification Effectiveness

**Figure 14:**
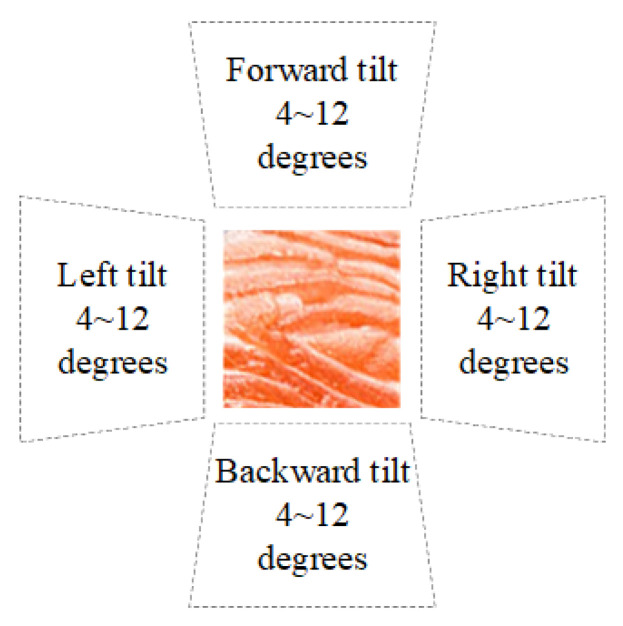
Schematic diagram of capture tilts at different directions and angles.

**Figure 15:**
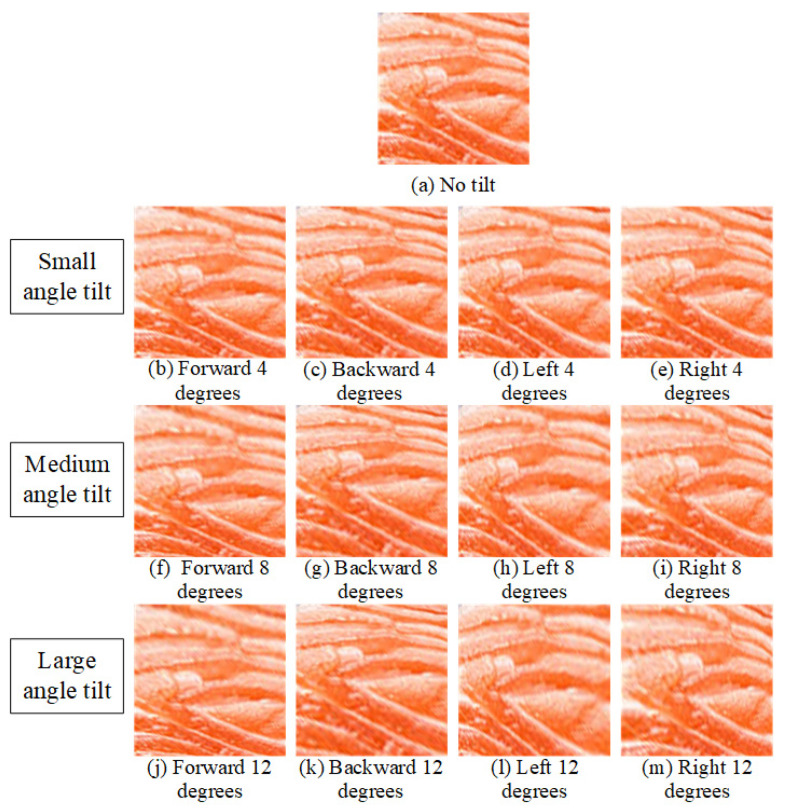
Sample images at different tilt directions and angles.

Consumers often capture tilted images in settings like restaurants or supermarkets. Figure 14 schematizes the image-capture tilt conditions examined in this study. A reference salmon flesh patch is shown at the center, surrounded by four wedge-shaped sectors indicating the tilt directions—forward, backward, left, and right. For each direction, three tilt magnitudes (4°, 8°, and 12°) are evaluated, producing 12 distinct imaging conditions. This diagram summarizes the controlled perspective deviations applied to simulate real-world capture bias (e.g., in restaurants or retail displays) and to quantify the system’s tolerance to camera tilt when assessing fish-meat classification and freshness. Corresponding sample images for each tilt direction and angle are shown in Figure 15.

**Figure 16:**
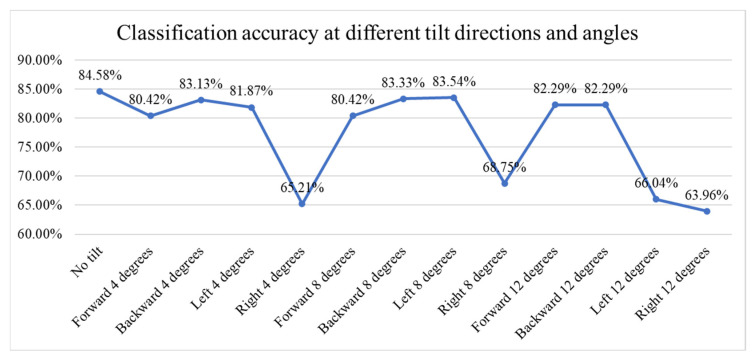
Line chart of the classification accuracy at different tilt directions and angles.

Figure 16 presents a line chart showing the classification accuracy of fish meat images under varying tilt directions and angles. The baseline accuracy without tilt is 84.58%. With small tilts (4 degrees), accuracy remains relatively high across most directions, except for a significant drop to 63.21% with a rightward tilt. Medium tilts (8 degrees) show stable performance in forward, backward, and left directions (above 80%), but, again, a notable drop occurs with right tilt (68.75%). At large tilts (12 degrees), accuracy remains above 82% for forward and backward directions but falls sharply for left (66.04%) and right (63.96%) tilts. Overall, the model is robust to forward/backward tilts up to 12° (≈2–4% loss) but is sensitive to rightward tilts and large left tilts (≈16–21 percentage-point loss).

### 4.7. Discussion and Limitations

Compared with state-of-the-art techniques such as hyperspectral imaging, near-infrared spectroscopy, or biochemical freshness assays, the proposed system provides a balanced compromise between accuracy and practicality. Laboratory-based methods, while offering objective chemical or microscopic validation, remain costly, resource-intensive, and inaccessible to everyday consumers. In contrast, the smartphone-based DenseNet121 approach relies solely on RGB images captured with widely available devices, enabling rapid, low-cost deployment without specialized equipment. This practicality is critical for consumer protection, as the tool empowers users to verify fish authenticity and freshness at the point of sale or consumption. By combining a simple interface with an efficient model, the system supports real-time decision making in contexts where mislabeling or fraud poses significant food safety risks.

The enhancements to DenseNet121, combined with transfer learning, make the model particularly well-suited for consumer-facing applications. The addition of global average pooling and dropout layers reduces overfitting and improves generalization, enabling the system to sustain high accuracy under real-world conditions such as motion blur, lighting variation, and camera angle shifts. Transfer learning further enhances practicality by freezing a portion of the layers, which shortens training time while preserving essential low-level feature representations. This dual benefit of efficiency and robustness ensures that the system delivers rapid, reliable results on resource-limited devices like smartphones, providing consumers with a trustworthy tool for fish meat classification and freshness grading.

While the proposed system demonstrates strong potential for automated fish meat classification and salmon freshness grading, several limitations should be noted. First, the dataset size is relatively small and partly sourced from online images, which may introduce bias due to uncontrolled variability in lighting and acquisition standards. Second, the labeling of freshness levels relied on manual expert annotation using the SalmoFan™ color chart. Although widely recognized in industry practice, this process is inherently subjective and is not validated through inter-rater agreement or biochemical testing. Third, the absence of objective biochemical indicators such as TVB-N, microbial load, or pH reduces the scientific robustness of the freshness grading results. Fourth, the dataset lacks external validation images captured in real-world scenarios such as supermarkets, restaurants, or consumer devices, which may limit the generalizability of the findings. Finally, the system has not yet been benchmarked against human inspection performance (both expert and non-expert), which would provide valuable context regarding its practical utility. Future research should address these issues by expanding the dataset, incorporating biochemical markers, collecting real-world validation images, and comparing machine predictions with human evaluations.

Another key limitation of this study is that the system has not yet been tested against advanced fraud techniques, such as artificially dyeing rainbow trout to resemble salmon. Although the model performs well on natural samples, deliberate pigment manipulation can obscure visual cues like color and marbling, reducing reliability. Addressing this requires future datasets to include dyed trout, along with complementary methods such as hyperspectral imaging, chemical validation, or anomaly detection, to enhance robustness and consumer protection.

To ensure a more reliable measure of performance consistency, rigorous statistical analysis is essential. In this study, performance metrics are reported as single values without cross-validation or confidence intervals. Although the observed numerical differences are large, future work will incorporate k-fold cross-validation (e.g., 5-fold) and statistical significance testing to verify that improvements are robust, generalizable, and not due to random variation.

The model’s interpretability also requires improvement. While the use of patch-based training partially reveals attention focus, a detailed visualization of decision making, using techniques like Grad-CAM, would help identify the key features the model uses, such as marbling, fat lines, or color shifts. This would build user trust and provide insight for further optimization. Lastly, while the model currently supports Atlantic salmon and rainbow trout classification, expanding its capabilities to include other fish species would require retraining and further validation. Future iterations should also aim to improve generalizability across camera devices, environmental settings, and fish preparation methods.

## 5. Conclusions

This study introduces a two-stage fish meat identification and freshness grading system based on an improved DenseNet121 architecture. By integrating global average pooling, dropout layers, and a streamlined classification pipeline, the model achieves high accuracy while remaining computationally efficient, making it suitable for consumer-oriented deployment via smartphones. Compared with one-stage and baseline models, the two-stage approach effectively reduces complexity and improves accuracy by sequentially addressing species classification and freshness grading.

Despite the promising results, the study has inherent limitations that must be addressed in future work. These include the relatively small dataset size, reliance on subjective manual labeling, absence of biochemical validation, and lack of external validation datasets. These factors may constrain generalizability and robustness under real-world conditions. In addition, the system’s performance has not been benchmarked against human inspectors, which would further validate its practical relevance.

Future work will focus on expanding and diversifying the dataset, integrating biochemical freshness markers to enhance scientific validity, and testing the model under real-world conditions such as packaged fish, supermarket lighting, and varied smartphone devices. External validation with consumer-captured images and benchmarking against human inspectors are also planned. Furthermore, the system has not yet been tested against deliberate fraud techniques such as artificially dyeing rainbow trout to mimic salmon, which may obscure genuine visual cues. To address this, future research will include such fraudulent cases and investigate complementary methods such as biochemical analysis and hyperspectral imaging to strengthen robustness and reliability.

In summary, mobile deployment is both feasible and essential, and further refinements in user experience, dataset diversity, and fraud detection capability will maximize the system’s societal impact as a practical tool for seafood authentication and consumer protection.

## Figures and Tables

**Table 1:** Appearance differences in Salmon flesh and rainbow trout flesh.

Name	Salmon	Rainbow Trout
Captured fillet images	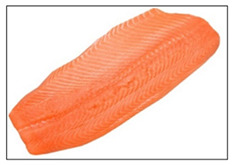	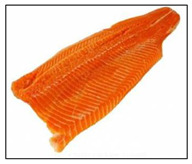
Appearance characteristics: 1. Color 2. Fat Marbling 3. Texture/Grain 4. Surface Sheen 5. Cut/Shape 6. Freshness Indicators	1. Vibrant pink to deep orange-red, depending on species. Wild salmon has richer hues; farmed salmon may be uniformly orange due to feed additives. 2. Pronounced white fat lines (intramuscular fat), especially in fattier cuts (e.g., belly or farmed salmon). Clear, evenly spaced streaks give a glossy, rich look. 3. Firm, smooth, and slightly glossy. Robust, compact grain with thicker, defined muscle fibers. Larger flake pattern when cut. 4. Glossy and moist due to higher fat content. Slightly oily shine, especially when fresh. 5. Thicker, robust fillets or steaks with smooth edges. Tapered fillet shape. Skin (if present) is silvery with a blue-green tint (wild) or uniform gray (farmed). 6. Fresh: Bright pink-orange to red-orange, glossy, no browning. Less Fresh: Faded color, less sheen, minor browning/yellowing at edges.	1. Pale pink to light orange, less vibrant. Often muted with a slightly grayish or whitish cast. 2. Minimal or faint white fat lines. Leaner appearance with subtle or no marbling. 3. Softer, delicate, less glossy. Finer grain with smaller, less distinct muscle fibers. Fragile flake pattern. 4. Matte or subdued surface. Less oily, may appear drier or less uniformly moist. 5. Thinner, more delicate fillets. Slimmer overall. Skin is often brownish with rainbow-like iridescent spots. 6. Fresh: Pale pink to light orange, slightly moist. Less Fresh: Fades to grayish/whitish, dry or sticky surface.

**Table 2:** Comparisons of the visual differences between Wild and Farmed Salmon flesh.

Visual Feature	Wild Salmon	Farmed Salmon
Fish meat	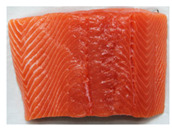	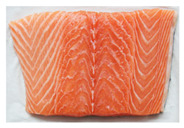
Color	Deep red/orange; natural gradient	Uniform light pink/orange via feed additives
Fat Marbling	Thin, sparse white lines	Prominent, thick white streaks
Texture	Firm, dense muscle structure	Softer, more buttery texture
Fillet Shape	Tapered, varied thickness	Uniform, thicker appearance
Sheen & Gloss	Matte, natural finish	Glossy, oilier due to higher fat

**Table 3:** Improved DenseNet121 with transfer learning architecture table.

Model Structure	TL (0%)	TL (10%)	TL (20%)	TL (30%)	TL (40%)	TL (50%)	TL (60%)	TL (70%)	TL (80%)	TL (90%)	TL (100%)
First 43 layers (10%)	Fine-tuning	Freezing	Freezing	Freezing	Freezing	Freezing	Freezing	Freezing	Freezing	Freezing	Freezing
First 85 layers (20%)	Fine-tuning	Fine-tuning	Freezing	Freezing	Freezing	Freezing	Freezing	Freezing	Freezing	Freezing	Freezing
First 128 layers (30%)	Fine-tuning	Fine-tuning	Fine-tuning	Freezing	Freezing	Freezing	Freezing	Freezing	Freezing	Freezing	Freezing
First 170 layers (40%)	Fine-tuning	Fine-tuning	Fine-tuning	Fine-tuning	Freezing	Freezing	Freezing	Freezing	Freezing	Freezing	Freezing
First 213 layers (50%)	Fine-tuning	Fine-tuning	Fine-tuning	Fine-tuning	Fine-tuning	Freezing	Freezing	Freezing	Freezing	Freezing	Freezing
First 256 layers (60%)	Fine-tuning	Fine-tuning	Fine-tuning	Fine-tuning	Fine-tuning	Fine-tuning	Freezing	Freezing	Freezing	Freezing	Freezing
First 298 layers (70%)	Fine-tuning	Fine-tuning	Fine-tuning	Fine-tuning	Fine-tuning	Fine-tuning	Fine-tuning	Freezing	Freezing	Freezing	Freezing
First 341 layers (80%)	Fine-tuning	Fine-tuning	Fine-tuning	Fine-tuning	Fine-tuning	Fine-tuning	Fine-tuning	Fine-tuning	Freezing	Freezing	Freezing
First 383 layers (90%)	Fine-tuning	Fine-tuning	Fine-tuning	Fine-tuning	Fine-tuning	Fine-tuning	Fine-tuning	Fine-tuning	Fine-tuning	Freezing	Freezing
First 426 layers (100%)	Fine-tuning	Fine-tuning	Fine-tuning	Fine-tuning	Fine-tuning	Fine-tuning	Fine-tuning	Fine-tuning	Fine-tuning	Fine-tuning	Freezing
Global average pooling	Modification	Modification	Modification	Modification	Modification	Modification	Modification	Modification	Modification	Modification	Modification
Activation function ReLU	Modification	Modification	Modification	Modification	Modification	Modification	Modification	Modification	Modification	Modification	Modification
Activation function Sofmax	Modification	Modification	Modification	Modification	Modification	Modification	Modification	Modification	Modification	Modification	Modification

**Table 4:** Characteristics of sample images for fish meat classification.

Category	Wild Salmon Steak (WS)	Wild Salmon Fillet (WF)	Farmed Salmon Steak (FS)	Farmed Salmon Fillet (FF)	Trout Meat (TM)	Other Meat (OM)
Experimental Image	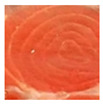	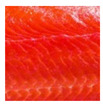	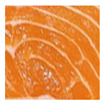	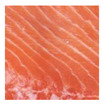	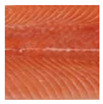	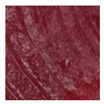
Description	Fat distribution is relatively unclear, with a circular pattern in the texture.	Fat distribution is relatively unclear, with parallel line patterns.	Fat distribution is more distinct, with circular texture patterns.	Fat distribution is more distinct, with parallel line texture patterns.	Flesh is relatively pale, with parallel line texture patterns.	Flesh is dark red, and fat distribution is unclear.

**Table 5:** Confusion matrix table for classification of fish species, growth patterns, and cutting methods.

	Predicted	Wild Steak (WS)	Wild Fillet (WF)	Farmed Steak (FS)	Farmed Fillet (FF)	Trout Meat (TM)	Other Meat (OM)	Total
Actual								
Wild Steak (WS)		*F*_1,1_	*F*_1,2_	*F*_1,3_	*F*_1,4_	*F*_1,5_	*F*_1,6_	$F_{1,\cdot }$= $\sum_{j=1}^{6}F_{1,j}$
Wild Fillet (WF)		*F*_2,1_	*F*_2,2_	*F*_2,3_	*F*_2,4_	*F*_2,5_	*F*_2,6_	$F_{2,\cdot }$= $\sum_{j=1}^{6}F_{2,j}$
Farmed Steak (FS)		*F*_3,1_	*F*_3,2_	*F*_3,3_	*F*_3,4_	*F*_3,5_	*F*_3,6_	$F_{3,\cdot }$= $\sum_{j=1}^{6}F_{3,j}$
Farmed Fillet (FF)		*F*_4,1_	*F*_4,2_	*F*_4,3_	*F*_4,4_	*F*_4,5_	*F*_4,6_	$F_{4,\cdot }$= $\sum_{j=1}^{6}F_{4,j}$
Trout Meat (TM)		*F*_5,1_	*F*_5,2_	*F*_5,3_	*F*_5,4_	*F*_5,5_	*F*_5,6_	$F_{5,\cdot }$= $\sum_{j=1}^{6}F_{5,j}$
Other Meat (OM)		*F*_6,1_	*F*_6,2_	*F*_6,3_	*F*_6,4_	*F*_6,5_	*F*_6,6_	$F_{6,\cdot }$= $\sum_{j=1}^{6}F_{6,j}$
Total		$F_{\cdot ,1}$= $\sum_{i=1}^{6}F_{i,1}$	$F_{\cdot ,2}$= $\sum_{i=1}^{6}F_{i,2}$	$F_{\cdot ,3}$= $\sum_{i=1}^{6}F_{i,3}$	$F_{\cdot ,4}$= $\sum_{i=1}^{6}F_{i,4}$	$F_{\cdot ,5}$= $\sum_{i=1}^{6}F_{i,5}$	$F_{\cdot ,6}$= $\sum_{i=1}^{6}F_{i,6}$	$F_{\cdot ,\cdot }$_._ = $\sum_{i=1}^{6}\sum_{j=1}^{6}F_{i,j}$

**Table 6:** Confusion matrix table for salmon freshness grading.

	Predicted	Pink Orange (PO)	Bright Orange (BO)	Red Orange (RO)	Total
Actual					
Pink Orange (PO)		*S*_1,1_	*S*_1,2_	*S*_1,3_	$S_{1,\cdot }$= $\sum_{j=1}^{3}S_{1,j}$
Bright Orange (BO)		*S*_2,1_	*S*_2,2_	*S*_2,3_	$S_{2,\cdot }$= $\sum_{j=1}^{3}S_{2,j}$
Red Orange (RO)		*S*_3,1_	*S*_3,2_	*S*_3,3_	$S_{3,\cdot }$= $\sum_{j=1}^{3}S_{3,j}$
Total		$S_{\cdot ,1}$= $\sum_{i=1}^{3}S_{i,1}$	$S_{\cdot ,2}$= $\sum_{i=1}^{3}S_{i,2}$	$S_{\cdot ,3}$= $\sum_{i=1}^{3}S_{i,3}$	$S_{\cdot ,\cdot }$= $\sum_{i=1}^{3}\sum_{j=1}^{3}S_{ij}$

**Table 7:** Comparison table of DenseNet121 and Improved DenseNet121 models.

Changed Items		DenseNet121	DenseNet121	Improved DenseNet121	Improved DenseNet121
Image augmentation	Scaling/Zooming	0.3	0.3	0.3	0.3
	Translation/Shifting	0.2	0.2	0.2	0.2
	Cropping	0.2	0.2	0.2	0.2
	Flipping/Mirroring	No	Yes	Yes	No
	Brightness adjustment	No	0.8~1.2	0.8~1.2	No
Main model structure		DenseNet121			
Classification layer	Global average pooling	No	No	Yes	Yes
	Dropout layer	No	No	0.2	0.2
	Dense	128, ReLU	128, ReLU	128, ReLU	128, ReLU
	Dropout layer	0.2	0.2	0.2	0.2
	Softmax	Softmax	Softmax	Softmax	Softmax
Performance	Accuracy	70.21%	70.83%	77.92%	84.58%

**Table 8:** Model effectiveness and efficiency changes across different methods for the fish meat classification stage.

	Classification Models	DenseNet121	Improved DenseNet121	Improved DenseNet121 + TL (40%)
Comparison Criteria				
Effectiveness: Accuracy (%)		70.83	84.58	81.04
Efficiency: Training time (s)		918	1533	1057
Compare with DenseNet121		-		
Change (%) in accuracy			19.41%	14.41%
Change (%) in training time			−66.99%	−15.14%
Compare with Improved DenseNet121		-	-	
Change (%) in accuracy				−4.18%
Change (%) in training time				31.05%

**Table 9:** Improved DenseNet121 model for salmon freshness grading: effectiveness and efficiency changes across different source domains.

	Source Domains	Random Weights	ImageNet Weights	Fish Meat TL (0%) Weights	Fish Meat TL (40%) Weights
Comparison Criteria					
Effectiveness: Accuracy (%)		73.75	70.83	73.75	72.50
Efficiency: Training time (s)		679	480	470	468
Compare with Random weights		-			
Change (%) in accuracy			−3.96%	0.00%	−1.69%
Change (%) in training time			29.31%	30.78%	31.08%
Compare with ImageNet weights		-	-		
Change (%) in accuracy				4.12%	2.35%
Change (%) in training time				2.08%	2.50%
Compare with Fish meat TL (0%) weights		-	-	-	
Change (%) in accuracy					−1.69%
Change (%) in training time					0.43%

**Table 10:** Accuracy comparison of classical machine learning methods and the Improved DenseNet121 model for salmon freshness grading.

Methods	Inputs	Overall Average	Wild Steak	Wild Fillet	Farmed Steak	Farmed Fillet
BPN	RGB + HSV components	62.50%	63.75%	55.00%	61.25%	70.00%
Fuzzy (FIS)		39.38%	40.00%	26.25%	46.25%	45.00%
ANFIS		61.75%	61.54%	69.62%	48.75%	67.09%
Improved DenseNet 121 with 0% freezing	RGB images	73.75%	78.33%	73.33%	73.33%	70.00%

**Table 11:** Comparison of sample size and experimental results of one-stage and two-stage approaches.

Approach	Two-Stage		One-Stage
Number of categories	Fish meat classification (6 categories)	Freshness grading (12 categories)	14 categories
Training images	160 images × 6 categories	40 images × 12 categories	70 images × 14 categories
Testing images	80 images × 6 categories	20 images × 12 categories	35 images × 14 categories
Accuracy			
(DenseNet 121)	(70.83%)	(None)	(52.25%)
Improved DenseNet 121	84.58%	73.75%	66.50%

**Table 12:** Images of six types of fish meat with three different blur levels.

Meat Types	Original	Mild Blur	Moderate Blur	Severe Blur
Wild steak	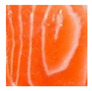	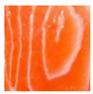	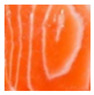	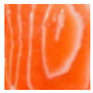
Wild fillet	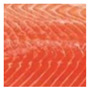	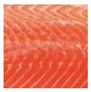	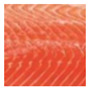	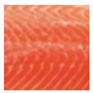
Farmed steak	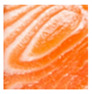	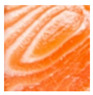	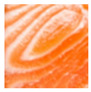	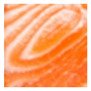
Farmed fillet	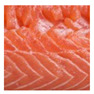	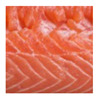	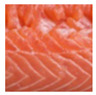	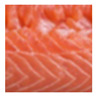
Trout meat	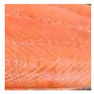	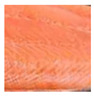	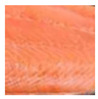	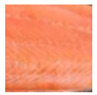
Other meat	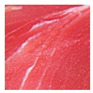	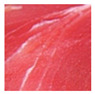	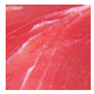	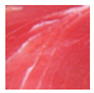

## Data Availability

The data will be made available upon request.

## Supplementary Materials

The following supporting information can be downloaded at: https://www.mdpi.com/article/10.3390/s25206299/s1, Figure S1. Roche SalmoFan fan. Figure S2. User interface of the classification and grading system developed in this study. Table S1. Number of training and testing images for fish meat classification. Table S2. Number of training and testing images for salmon freshness grading.
