# Supplementary material for: A Deep Learning-Based Sensing System for Identifying Salmon and Rainbow Trout Meat and Grading Freshness for Consumer Protection
Source: Sensors (Basel). 2025 Oct 11;25(20):6299. doi: 10.3390/s25206299 (PMC12567205; doi:10.3390/s25206299)
Supplement: Supplementary file 1 [file sensors-25-06299-s001.zip › sensors-3880111-supplementary.pdf]

Supplementary Material

Supplementary Figure S1:

Roche SalmoFan™ color scale, a physical fan with numbered color chips (usually ranging from 20 to 34), is used to adjust feed formulations based on customer preferences.

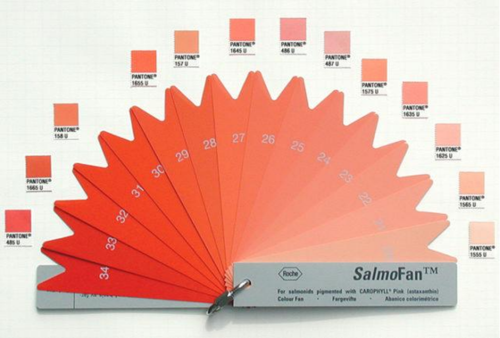

Supplementary Figure S1. Roche SalmoFan fan.

Supplementary Figure S2:

The user interface of the automated fish meat classification and salmon freshness grading system developed in this study. On the left, the parameter settings panel allows users to configure model training parameters. Below this, performance indicators and average processing time per image are presented. The central and right portions illustrate the six-step workflow. The system classifies the fish meat as “Farmed salmon fillet” and grades its freshness as “Fresh Orange,” as indicated in the final output section. It supports usability and transparency in automated seafood quality assessment.

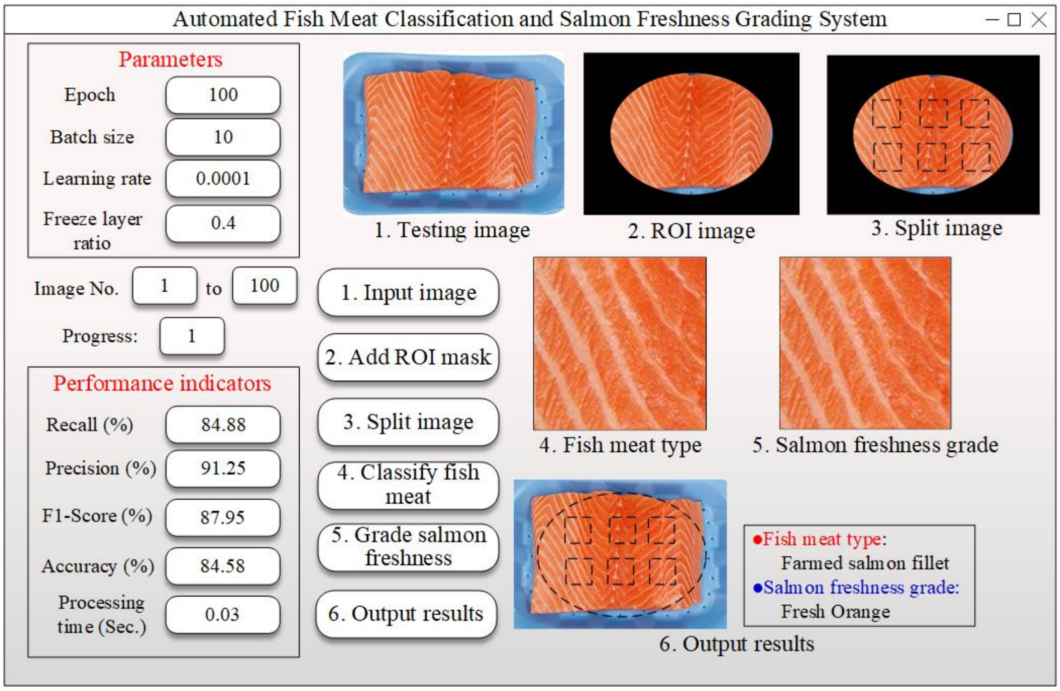

Supplementary Figure S2. User interface of the classification and grading system developed in this study.

Supplementary Table S1.

Dataset composition for fish meat classification, showing the number of training and testing images across all categories.

Supplementary Table S1. Number of training and testing images for fish meat classification.

	Wild Steak (WS)	Wild Fillet (WF)	Farmed Steak (FS)	Farmed Fillet (FF)	Trout Meat (TM)	Others Meat (OM)	Total
Training	160	160	160	160	160	160	960
Testing	80	80	80	80	80	80	480
Total	240	240	240	240	240	240	1440

Supplementary Table S2.

Dataset composition for salmon freshness grading, presenting the distribution of training and testing images across the three freshness levels.

Supplementary Table S2. Number of training and testing images for salmon freshness grading.

	Wild Steak (WS)		Wild Fillet (WF)		Farmed Steak (FS)		Farmed Fillet (FF)	
	Training	Testing	Training	Testing	Training	Testing	Training	Testing
Pink Orange (PO)	40	20	40	20	40	20	40	20
Bright Orange (BO)	40	20	40	20	40	20	40	20
Red Orange (RO)	40	20	40	20	40	20	40	20
Sum	120	60	120	60	120	60	120	60
	180		180		180		180	
	720							
